# Differential expression of snoRNAs in oral squamous cell carcinomas: new potential diagnostic markers

**DOI:** 10.1080/14756366.2018.1426574

**Published:** 2018-01-26

**Authors:** Cintia Chamorro-Petronacci, Mario Perez-Sayáns, Maria Elena Padín-Iruegas, Xabier Marichalar-Mendia, Mercedes Gallas-Torreira, Abel García García

**Affiliations:** a Oral Medicine, Oral Surgery and Implantology Unit, Faculty of Medicine and Dentistry, Instituto de Investigación Sanitaria de Santiago (IDIS), Santiago de Compostela, Spain;; b Anatomy and Human Embryology, Functional Biology and Health Science Department, Vigo University, Vigo, Spain;; c Faculty of Medicine and Dentistry, Stomatology Department, Universidad del Pais Vasco/Euskal Herriko Unibertsitatea, Bilbao, Spain

**Keywords:** Oral cancer, epigenetics, snoRNA

## Abstract

**Background:** Small nucleolar RNAs (snoRNAs) are small non-coding RNA sequences whose most studied function is ribosome biogenesis. The altered expression of snoRNA is observed in tumoral processes such as breast cancer and multiple myeloma. However, we have not found any references to snoRNAs in oral squamous cell carcinomas (OSCC) in the literature at the time this article was written.

**Material and methods:** We have analyzed snoRNA expression in frozen OSCC tissue samples and have compared them to healthy controls. RNA was extracted from a total of eight OSCC samples and eight control samples, measuring the differential expression of small RNAs with the Affymetrix^®^ miRNA 4.1 Array Plate microarray platform.

**Results:** Results were analyzed using the Transcriptome Analysis Console 3.0 (TAC) software. We obtained a total of 16 deregulated snoRNAs of which one was over expressed and 15 were under expressed. SnoRNAs expression was altered in OSCC and could serve as a diagnostic marker.

## Introduction

SnoRNAs (small nuclear RNAs) are a large class of non-coding RNA found, in sequences of eukaryotic cells. The main functions under study in this type of small non-coding RNAs is ribosome biogenesis, more specifically, the components of ribonucleoprotein complexes (snoRNP)^1^. snoRNAs are divided into two groups, C/B and H/ACA box, which differ in terms of characteristics and structure, as well as other protein union points and the chemical modifications they catalyze^2^.

The function of the snoRNA C/D and H/ACA box is developed in preribosomal RNA, modifying nucleotides at specific locations. The members of the C/D box are involved in 2’-O-methilation and those of the H/ACA box in pseudouridylation.

New snoRNAs are identified through ultrasequencing and immunoprecipitation by the union of RNA to certain proteins, although the lack of association of some snoRNAs and ribonucleic complexes in the reviewed studies lead us to believe that the regulatory functions they develop, may differ from those involved in ribosomal biogenesis. Some studies suggest that snoRNAs can also participate in the regulation of alternative splicing and posttranscriptional modifications of messenger RNA^3^.

Therefore, alterations in the expression of these RNAs can affect several vital and pathological processes, becoming a target for the treatment and diagnosis of human neurodegenerative, oncological, or viral pathologies^4^. The altered expression of snoRNAs has been observed in cancer processes such as breast cancer and multiple myeloma^5^^,^^6^. Specifically, the increased expression of ACA11 seems to produce resistance to chemotherapy and increased proliferation of multiple myeloma cell lines^5^.

In our bibliographic search, we found no references of snoRNA expression in oral squamous cell carcinomas (OSCC). OSCC is the most frequent malignancy of the oral cavity and the prognosis of OSCC patients has not improved in the past years, despite the advances in the biological and technological knowledge^7^. Only a fraction of the patients exposed to risk factors (tobacco, alcohol, and human papilloma virus) develops OSCC^8^. This suggests that there are other factors, known or unknown, that we are not considering in oral cancer tumorigenesis.

Due to this need to continue to increase our knowledge on OSCC and thus improve the survival of patients, our project seeks to determine the expression of snoRNA in samples of frozen OSCC tissue and compare them to healthy tissue samples using microarray technology.

## Materials and methods

### Samples

We selected OSCC samples from the buccal mucosa, the soft palate, the retromolar area, the tongue, and the floor of the mouth, all of which had been extracted by the Maxillofacial Surgery Unit of Complejo Hospitalario Universitario de Santiago de Compostela (Santiago Teaching Hospital), which amounted to eight cases that had been preserved at −80 °C at the hospital’s biobank (SERGAS), from 1998 to 2015. The same service also obtained eight control samples of keratinized gums, collected in 2015 from the patients that visited the service for the extraction of their third molar.

All the samples were stored at the Santiago de Compostela Biobank (SERGAS). Optimal cutting temperature freezing cuts were developed on each sample, 14 cuts in 14 micrometers.

This research has obtained the approval of the Clinical Research Ethics Committee of Galicia Ref. 2015/132 (CEIC), currently known as the “Comité Autonómica da Ética da Investigación” (CAEI). All procedures were carried out with the adequate understanding and written consent of the subjects.

### Total RNA extraction and microarray

Total RNA was extracted using the el mirVana^TM^ miRNA isolation kit. The concentration of the samples was measured with NanoDrop 2000 (Thermo Fisher Scientific, Waltham, MA) and their integrity was assessed (RIN) using NanoChip 6000 and bioanalyzer. Once total RNA was extracted, we used Affymetrix^®^ FlashTag^TM^ Biotin HSR RNA Labeling Kit to mark the samples. The microarray platform we used was Affymetrix^®^ miRNA 4.1 array plate that contained enough probes to identify 2578 human miRNAs and 1996 human snoRNAs and scaRNA (“small Cajal body-specific RNA”). The analysis of the differential expressions was developed using the software Transcriptome Analysis Computer 3.0 by Affymetrix (free access). Last of all, deregulated snoRNAs were searched on the snoRNA-LMBE-db data base (https://www-snorna.biotoul.fr/index.php, December 2016). All methods were performed in accordance with the relevant guidelines and regulations.

### Statistical analysis

For the univariate description we used basic descriptive statistics: mean, standard deviation, frequency, and percentage.

For the differential expression of snoRNAs, the Transcritpome Analysis Console 4.0 (TAC) developed a univariate ANOVA analysis selecting genes with a “fold change*”* of over ±2 and a *p* valueunder .05.

To perform the comparison between clinical features and snoRNA expression, we have recoded the expression values in semiquantitative variables, considering over expressed when the expression was over the mean expression level and under expressed when the expression was under the mean expression level. The relationship between clinical and pathological parameters and the expression of snoRNAs deregulated were calculated using non-parametric statistics with U Mann Whitney test and parametric chi-square test depending on the application conditions.

Kaplan Meier analysis and the log-rank test were performed to identify survival differences in OSCC patients. Differences were considered statistically significant when *p* was less than .05.

## Results

### Descriptive analysis

The mean age of the eight cases was 60.75 years, with a range of 41–75 years. Of the eight cases, five were women (62.5%) and three were men (37.5%). Of the eight primary intraoral tumors, three were on the tongue (37.5%), two on the gum (25%), one1 in the molar trigone area (12.5%), one in the floor of the mouth (12.5%), and one in the buccal mucosa (12.5%). Two were stage II at the time of surgery and six were stage IV. Of the eight cases, two were smokers (25%), one was a former smoker (12.5%), and five were nonsmokers (62.5%).The mean age of the controls was 37.75 years, with a range of 18–75 years. Of the eight controls, two were women (25%), and six were men (75%). In the control group, two were smokers (25%) the other six were nonsmokers (75%).

The mean concentration of total extracted RNA in the case group was 1145.25 ng/µL and in the control group 785.33 ng/µL. The mean RIN of the total extracted RNA in the case group amounted to 8.275 and 8.15 in the control group.

### SnoRNA differential expression in OSCC

A total of 16 snoRNAs presented a significantly different expression in case samples in contract with the control samples; 15 were overexpressed and one was under expressed. Table 1 shows the name of the deregulated snoRNAs with their fold change and *p* values. Each snoRNA was searched on the snoRNA-LMBE-db database, from which we collected information on each of their families, their location, length in nucleotides, and function (Table 2).

**Table 1.** Deregulated snoRNAs comparing the samples of the cases and the controls, with their fold change and *p* values.

snoRNA	Fold change	*p* value
14qI-4	−3.30	.000777
14qII-22	−2.21	.009243
ACA17	−2.32	.014394
ENSG00000263442	−2.08	.017241
ENSG00000264591	−2.08	.017241
ENSG00000265325	−2.08	.017241
ENSG00000265607	−2.08	.017241
ENSG00000266646	−2.08	.017241
ENSG00000266755	−2.08	.017241
U84	−2.08	.017241
mgh18S-121	−2.84	.031249
U18A	−2.29	.034712
U8	−2.02	.036967
ENSG00000207118	3.03	.038715
14qII-12	−2.65	.044976
U28	− 2.37	.046883

**Table 2.** Deregulated snoRNAs in OSCC specifying the type of snoRNA, the family they belong to, the chromosome in which they are located, the nucleotides forming it and their function, known or unknown (?).

Name	Family	Location	Length (nt)	Function
ACA17	H/ACA box	Chromosome 9	133	It is believed that pseudouridylation of U4659, U4598, and U4937 waste and the ribosomal subunit 28S rRNA
14q1-4	C/D box	Chromosome 14, intronic sequence of MEG8	74	?
14q11-12	C/D box	Chromosome 14, intronic sequence of MEG8	74	?
14q11-22	C/D box	Chromosome 14, intronic sequence of MEG8	71	?
U84	C/D box	Chromosome 19, intronic sequence of BAT1	78	?
mg18S-121 (Z17A)	C/D box	Chromosome 17, intronic sequence of RPL23A	72	Its function is to guide 2’O-ribose methylation of 18S rARN U121.
U18A	C/D box	Chromosome 15, intronic sequence of ribosomal protein RPL4	70	Its function is to guide 2’O-ribose methylation of 28S rARN A1313
U8	C/D box	Independent Transcription Unit	136	?
U28	C/D box	Chromosome 11, intronic sequence of gene U22	75	Methylation guide of 2’O-ribose in 18S rARN C1391.
ENSG00000263442	C/D box	Chromosome 6	78	?
ENSG00000264591	C/D box	Chromosome 6	78	?
ENSG00000265325	C/D box	Chromosome 6	78	?
ENSG00000265607	C/D box	Chromosome 6	78	?
ENSG00000266646	C/D box	Chromosome 6	78	?
ENSG00000266755	C/D box	Chromosome 6	78	?
ENSG00000207118	C/D box	Chromosome 11	87	?

### Associations with clinical and pathological features

We examined the expression of each snoRNA with clinical features and we have found that 14qII-22 have statistical significant different expression between gender categories (*p* = .031). Women have an average expression of 4.93 (SD = 0.95) whereas men have an average of 3.32 (SD = 1.43). However, there were no significant correlation of snoRNAs expression with other clinical features such as age or tobacco consumption.

Further, we have analyzed the pathological prognostic parameters in patients with OSCC and expression levels and results showed that U28 low expression is correlated with a good differentiation (*p* = .018). Three of the cases (75%) with low expression had good differentiation versus one case with bad differentiation. We have found no correlation between other pathological features as localization, TNM stage radiotherapy or chemotherapy, and snoRNA expression.

### Survival analysis

The average of OSCC patient’s survival time was 50.17 months (CI 95%: 2.539–97.795). We performed Kaplan-Meier curves for overall survival by comparing the patients with high and low snoRNA expression. Those patients which ENSG000002645191, ENSG00000263442, ENSG00000265325, ENSG00000265607, ENSG00000266646, ENSG00000266755, ENSG00000207118, U8, and U28 expression was under the mean expression level had a higher survival time (74.47 versus 25.89 months). Those patients which U18A, 14qII-12 expression was over the mean expression level had a higher survival time. Although the differences were not statistically significant. Both patients who had died, had an expression of mgh18S-121 and ACA17 under the mean expression level. On the other hand, the expression of 14qII-22 of the same patients was over the mean expression level, although these differences were not statistically significant.

## Discussion

The results of this study reveal that the expression of snoRNAs in OSCC is different when compared to healthy tissue, which makes them a possible diagnostic and prognostic markers in OSCC. The information of each deregulated snoRNA was searched on the snoRNA-LMBE-db database^9^. We have not found information regarding the function or the possible targets for most of the deregulated snoRNAs in this study. Abnormal expression of snoRNA hasn't been studied in OSCC although it has been analyzed in different types of cancer such as leukemia^10^, colorectal^11^, and pancreatic cancer^12^. In our revision of McManhon et al. we found that they reported on the differential expression of snoRNAs in different diseases including head and neck cancers, finding SNORA71C, which belongs to the H/ACA family, to be under expressed. Deregulated snoRNAs in our study did not match the ones collected in this revision^13^.

Altered expression of non-coded RNAs in OSCC has been studied with the purpose of finding diagnostic and prognostic markers and therapeutic targets^14^. However, the results of this research have been different and on occasions, contradictory^15^. The interpretation of the data of deregulated snoRNAs requires further research in the field, since small fragments of RNA have been found in the C/D and H/ACA box with traditional blocking function, similar to microRNAs and have been associated to Argonaute 2 (AGO2). The Argonaute protein is necessary for RNAs, such as microRNAs, to form the RISC complex, and thus induce the silencing of messenger RNAs. Therefore, a series of microRNAs that silence messenger RNAs and are originated from precursors leading to the C/D or H/ACA boxes have been discovered. These microRNAs match the snoRNAs, as for example, SNORD12b/HBII-99B (miR-1259), SNORD126 (miR-1201), SNORA34/ACA34 (miR-1291), SNORA81/HBI-61 (miR-1248), and SNORA36B/ACA36B (miR-664)^2^. None of these snoRNAs have shown a deregulated expression in our study.

Considering the location of the deregulated snoRNAs in this study, an alteration in the expression is expected to be caused by mutations in the chromosomal regions. Upon review of the literature on alterations and chromosomal instability in OSCC, some studies reveal that in chromosome 9, where ACA17 is located, there are mutations in almost 70% of OSCC cases, affecting the function of proteins such as p16^16^. Genetic alterations that affect proteins in chromosome 11 have also been reported, such as Cyclin D1, which has been broadly studied in tumor processes^17^.

We have used keratinized gum as the healthy control tissue because there were OSCC samples from gum also, it is a location that also can suffer OSCC, it is exposed to risk factors in a similar way as the other parts of the oral cavity and it was the less invasive manner to take the control of the healthy patients. In a future, it would be better to use healthy samples of the same type of tissue (buccal mucosa, soft palate, retromolar region, tongue, and floor of the mouth) thus matching the location of the OSCC samples.

Although the mean age of the cases group doubled the mean age of the control group, we have found no references in the literature regarding the alteration of snoRNA expression in relation to age.

Our results have revealed different 14qII-22 expression levels between males and females and also have found lower U28 expression levels in those cases with good differentiation. Although we have found no references in literature that correlates snoRNA expression and clinical features and prognosis, to verify the usefulness of these snoRNAs as diagnostic and prognostic markers in OSCC it is necessary to corroborate the results by means of Quantitative reverse transcription -polymerase chain reaction and study a larger sample size.

The results of our study are a first step in future research on the role of snoRNAs in oral cancer.
